# Cloning and Expression Analysis of Two Kdm Lysine Demethylases in the Testes of Mature Yaks and Their Sterile Hybrids

**DOI:** 10.3390/ani10030521

**Published:** 2020-03-20

**Authors:** Zhenhua Shen, Lin Huang, Suyu Jin, Yucai Zheng

**Affiliations:** College of Life Science and Technology, Southwest Minzu University, Chengdu 610041, China; zhenhuashen91@hotmail.com (Z.S.); huanglin200426@hotmail.com (L.H.); syjin65@163.com (S.J.)

**Keywords:** epigenetics, cattle–yak, male sterility, KDM1A, KDM4B, H3K36me3

## Abstract

**Simple Summary:**

The hybrid of male cattle (*Bos taurus*) with female yaks (*Bos grunniens*) is called the cattle–yak. All female cattle–yaks are fertile, but all males are sterile. To date, there is no clear conclusion on the mechanism leading to cattle–yak male sterility. The domain conservation and expression profiles of lysine histone demethylases (KDMs) suggest that they might play important roles during gametogenesis. The objective of this study was to explore the molecular mechanism for male sterility of yak hybrids based on two demethylases, *KDM1A* and *KDM4B*. The mRNA and protein expression of *KDM1A* and *KDM4B* were dramatically decreased in the testes of adult cattle–yaks compared with adult yaks. In addition, the level of H3K36me3 in the testes of cattle–yaks was significantly lower than in yaks. These results suggest that the male sterility of cattle–yaks might be associated with reduced histone methylation modifications. These results provide valuable epigenetic information regarding the molecular mechanism resulting in male sterility of cattle–yaks.

**Abstract:**

The objective of this study was to explore the molecular mechanism for male sterility of yak hybrids based on two demethylases. Total RNA was extracted from the testes of adult yaks (*n* = 10) and yak hybrids (cattle–yaks, *n* = 10). The coding sequences (CDS) of two lysine demethylases (KDMs), KDM1A and KDM4B, were cloned by RT-PCR. The levels of *KDM1A* and *KDM4B* in yaks and cattle–yaks testes were detected using Real-time PCR and Western blotting for mRNA and protein, respectively. In addition, the histone methylation modifications of H3K36me3 and H3K27me3 were compared between testes of yaks and cattle–yaks using ELISA. The CDS of *KDM1A* and *KDM4B* were obtained from yak testes. The results showed that the CDS of *KDM1A* exhibited two variants: variant 1 has a CDS of 2622 bp, encoding 873 amino acids, while variant 2 has a CDS of 2562 bp, encoding 853 amino acids. The CDS of the *KDM4B* gene was 3351 bp in length, encoding 1116 amino acids. The mRNA and protein expression of *KDM1A* and *KDM4B,* as well as the level of H3K36me3, were dramatically decreased in the testes of cattle–yaks compared with yaks. The present results suggest that the male sterility of cattle–yaks might be associated with reduced histone methylation modifications.

## 1. Introduction

Yak (*Bos grunniens*) is one of the main livestock species raised on the Qinghai–Tibet Plateau in China. It has the ability to adapt to an environment of high altitude, low temperatures, and hypoxia. Yak milk and milk products are the main dietary component of Tibetan people as well as an important source of income for local families [1]. The cattle–yak, an interspecific hybrid of yaks and cattle, has the characteristics of higher milk and meat yield. At the same time, cattle–yak can adapt to the plateau environment. Interestingly, female cattle–yaks are fertile, while the males have been found to be sterile [2]. In previous studies, the main reason for male cattle–yaks sterility was inability to correctly form the synaptonemal complex during spermatogenesis [3,4]. There are many genes involved in the process of spermatogenesis has been studied, including the *Hsp27*, *PIWIL1*, *Mei1*, *Dmrt7*, *SYCP3*, *Cdc2*, and *Cdc25A*. These genes have lower mRNA levels in cattle–yak testes than in those of their parental yaks and cattle [4,5,6,7,8,9,10]. To date, there is no clear understanding regarding the mechanism of cattle–yak male sterility.

Epigenetics is characterized by RNA interference, chromatin remodeling, DNA methylation, and histone modifications, which are important regulators in a number of biological processes [11]. Epigenetics also plays important roles in spermatogenesis and meiosis, and histone modifications are involved in these processes [12,13,14]. Histones are chromosomal proteins and serve as the structural unit for packaging of DNA. Amino acid residues at the N-terminus of histones can be methylated, acetylated, phosphorylated or ubiquitinated [15]. Methylation occurs at the N-terminal arginine and lysine residues, and these lysine residues can be monomethylated, demethylated or trimethylated. The functional consequences of histone lysine methylation can be associated with either transcriptional activation or repression, depending on the specific lysine residue that is modified and the degree of methylation [15,16,17]. Post-translational modification of histones is an essential mechanism for regulating gene expression.

Mammalian spermatogenesis is a complex process which can be divided into three phases: spermatocytogenesis, meiosis, and spermiogenesis [18]. Methylation is an important form of histone modification and has been shown to be essential for spermatogenesis, including both addition and removal of methylation by some methyltransferases and demethylases, respectively [14,19]. Research showed that H3K4me2/3, H3K27me3, and H4K20ME1/2/3 are dynamically distributed throughout the various stages of spermatogenesis [12]. G9a is a methyltransferase that monomethylates and dimethylates H3K9, which then mediates epigenetic gene silencing and is pivotal for meiotic prophase progression [20]. The H3K9me1 and H3K9me2 histone demethylase JMJD1A is essential for spermatogenesis [21]. Previous studies have suggested that it is important to consider the epigenetic perspective when conducting research on cattle–yak spermatogenesis aiming to explore the mechanisms of cattle–yak sterility [5,22,23].

The domain conservation and expression profiles of KDM histone demethylases suggest that they might play important roles during gametogenesis [24]. A previous study discovered that the active gene associated with H3K36 trimethylation (H3K36me3) is related to germ cell differentiation [25]. The genes for H3K27me3 were involved in the processes of spermatogenesis and germ cell development. The profiles of trimethylated H3K27 (H3K27me3) in spermatozoa of water buffalo bulls differ between highly fertile and sub-fertile buffalo bulls [26]. Due to the significant role of lysine demethylases in spermatogenesis, it is hypothesized that the sequences and expression levels of *KDM1A* and *KDM4B* might be associated with male cattle–yak sterility. The objective of this research is to explore the possible relationship between epigenetics and male cattle–yak sterility by comparing the patterns of demethylase gene expression and histone methylation. In this study, the CDS of yak *KDM1A* and *KDM4B* were cloned, followed by a comparison of *KDM1A* and *KDM4B* mRNA and protein levels in the testes of sterile cattle–yaks and normal yaks. We then compared the histone methylation modifications of H3K36me3 and H3K27me3 between yaks and cattle–yaks.

## 2. Materials and Methods

### 2.1. Animals and Sampling

All experimental male Maiwa yaks (*Bos grunniens*, *n* = 10) and male cattle–yaks (F1 hybrids between male cattle and female Maiwa yaks, *n* = 10) were provided by a slaughterhouse in Qingbaijiang, Sichuan Province, China. The slaughtered yaks and cattle–yaks were around 4–6 years old. All yaks and cattle–yaks are raised in the same high-altitude environment and grazed on natural grassland. The testes and epididymes were collected and cut into several parts. All testes were promptly frozen in liquid nitrogen. A part of the epididymis was promptly frozen in liquid nitrogen, and other parts were fixed in fixation solution (Bouin’s solution). The epididymes of sexually mature yaks and cattle–yaks were made into paraffin sections, and the anatomical structure of the epididymis was observed under a microscope after H&E staining. All experiments were conducted in accordance with the Regulation on the Administration of Laboratory Animals (2017, China State Council).

### 2.2. Histological Comparison of Yak and Cattle–Yak Epididymes

The epididymes of yaks and cattle–yaks were fixed overnight in Bouin’s solution, then dehydrated and embedded in paraffin and sectioned at 4 μm standard, followed by hematoxylin and eosin (H&E) staining using standard techniques. The sections were observed under an optical microscope (Precipoint_M8, Freising, Germany).

### 2.3. RNA Extraction and cDNA Synthesis

Total RNA was isolated from the testes of yaks and cattle–yaks with TRIzol reagent (Invitrogen, Carlsbad, CA, USA) according to the manufacturer’s instructions. The reverse transcription was performed using the Revert Aid First Strand cDNA Synthesis Kit (Thermo, Waltham, MA, USA) according to the manufacturer’s instructions. total RNA, Oligo (dT)18 primer and Random Hexamer primer, water(nuclease-free), 5× Reaction Buffer, RiboLock RNase Inhibitor, 10 mM dNTP Mix, RevertAid M-MuLV RT were used in first strand cDNA synthesis. The PCR program was as follow: Incubate for 5 min at 25 °C followed by 60 min at 42 °C. Terminate the reaction by heating at 70 °C for 5 min. The products were used for cloning and expression analysis of *KDM1A* and *KDM4B*.

### 2.4. Cloning and Sequencing of the KDM1A and KDM4B Genes of Yaks

All PCR primers and conditions used in this study are shown in Table 1. *KDM1A* and *KDM4B* were cloned under similar conditions. Each PCR reaction was performed using Super Taq DNA polymerase (GeneCopoeia, Rockville, MA, USA). The PCR conditions were 94 °C for 5 min; 35 cycles of 94 °C for 30 s, 59 °C for 30 s, and 72 °C for 4 min; 72 °C for 7 min. The PCR products were separated using 1% agarose gel electrophoresis and purified using a DNA purification kit (Omega, Norcross, GA, USA). The purified products were cloned into the pMD19-T vector (TaKaRa, Shiga, Japan). The ligate was transformed into competent *Escherichia coli* DH5α cells (TIANGEN, Beijing, China). *KDM1A* and *KDM4B* had twelve and three positive clones, respectively, which were sequenced by Sanger sequencing at Chengdu Tsingke (Chengdu, China).

### 2.5. Analysis of KDM1A and KDM4B mRNA Expressions by Quantitative Real-time PCR

Quantitative Real-time PCR was used to compare the mRNA levels of *KDM1A* and *KDM4B* in the testes of adult yaks (*n* = 10) and adult cattle–yaks (*n* = 10). The primers are listed in Table 1. GAPDH and 18S RNA were used as reference genes [27]. The Real-time PCR reactions were performed using TB Green™ Premix Ex Taq™ II (TaKaRa, Shiga, Japan). The reactions contained 2 μL cDNA, 12.5 μL TB Green Premix Ex Taq II, 1 μL for each primer (from a 10 μmol/L stock), and the addition of ultra-pure water to make the volume up to 25 μL. The PCR was performed with the CFX96 Real-Time PCR Detection System (Bio-Rad, Hercules, CA, USA). The PCR conditions for *KDM1A*, *KDM4B*, and *GAPDH* were one cycle of 30 s at 95 °C followed by 40 cycles of 5 s at 95 °C and 1 min at 60 °C. The PCR conditions for *18S RNA* were set as the following: one cycle of 1 min at 95 °C followed by 40 cycles of 15 s at 95 °C, 20 s at 59 °C, and 30 s at 72 °C. The melting curve was analyzed from 65 to 95 °C by with plate readings taken at each 0.5 °C increment. Each sample in the analysis was technically duplicated.

### 2.6. Analysis of KDM1A and KDM4B Protein Expression by Western Blotting

The testicular tissues were lysed in RIPA buffer (150 mM NaCl; 50 mM Tris-Cl, pH 8; 1% NP-40; 0.5% deoxycholate; 0.1% SDS) (Solarbio, Beijing, China) with phenylmethylsulfonylfluoride (PMSF, final concentration 1 mM) (Solarbio, Beijing, China). The resulting samples were separated using 10% SDS-PAGE and transferred to PVDF membranes. After blocking for 1 h in 5% non-fat milk in TBST at 25 °C, the membranes were incubated with specific primary antibodies overnight at 4 °C. The primary antibodies used were KDM1A (Novus, Centennial, CO, USA, 1:1000), KDM4B (Abcam, Cambridge, UK,1:2000), β-tubulin (Zen Bioscience, Chengdu, China, 1:5000). The primary antibody binding was visualized with horseradish peroxidase-conjugated goat anti-rabbit or anti-mouse IgG (1:10,000, ZSGB-BIO, Beijing, China) for 1 h at 25 °C. The signal intensities were measured using Chemiluminescent HRP Substrate (Millipore, Billerica, Massachusetts, USA) and image analysis software (ImageJ1.8.0, NIH, Bethesda, MD, USA).

### 2.7. Quantification of H3K36 and H3K27 Trimethylation by ELISA 

Total histones were extracted from the frozen testes of yaks and cattle–yaks using the EpiQuik™ Total Histone Extraction Kit (Epigentek, Farmingdale, NY, USA) according to the manufacturer’s instructions. Quantification of trimethylated H3K36 and H3K27 was performed using the EpiQuik global trimethyl histone H3K36 and H3K27 quantification kit (Epigentek, Farmingdale, NY, USA) following the manufacturer’s protocol. The absorbance was read using a microplate reader at a wave length of 450 nm. The amount of trimethylated H3K27 or H3K36 was calculated according to the manufacturer’s instructions for the kits.

### 2.8. Statistical Analysis

The relative quantities of the reference genes GAPDH and 18S RNA were determined, and a normalization factor was calculated based on the geometric mean for internal normalization. The threshold cycle (Ct) resulting from RT-PCR was analyzed using the 2^-ΔΔCt^ method [27]. All data were analyzed using GraphPad Prism 8.01 (GraphPad Software, San Diego, CA, USA). Values and expressed as mean ± SD. All the data were performed normal distribution analysis by Anderson-Darling test (alpha = 0.05). In these data, mRNA and protein levels of KDM1A were not normally distributed and performed non-parametric Mann-Whitney tests. All the other data are normally distributed and performed two-tailed Student’s t-test. Significant differences were considered to exist when the *p*-value was less than 0.05.

## 3. Results

### 3.1. H&E-Stained Sections of Epididymes from Yaks and Cattle–Yaks

**Figure 1:**
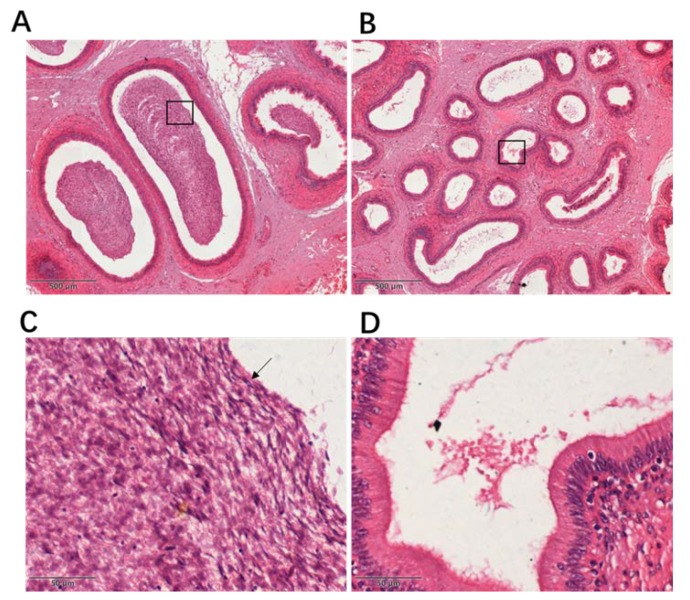
Histological examination of the epididymis in yaks and cattle–yaks. Magnification: 40× (images A, B), 400× (images C, D). (**A**) Sections of H&E-stained yak epididymis. (**B**) Sections of H&E-stained cattle–yak epididymis. (**C**) Enlargement of the part of image A framed by the black rectangular box. The arrow indicates the yak sperm. (**D**) Enlargement of the part of image B framed by the black rectangular box.

A large amount of sperm can be found in the epididymal ducts of the yaks (Figure 1A,C). However, in the epididymis sections of cattle–yaks, no sperm was observed in the epididymal ducts (Figure 1B,D).

### 3.2. Cloning and Sequencing of the Yak KDM1A and KDM4B Genes

The CDS of the *KDM1A* and *KDM4B* genes of yaks were cloned from yak testes. The sequencing results of *KDM1A* revealed two variants: variant 1 has a CDS of 2622 bp (GenBank accession no. MK411808), encoding 873 amino acids, while variant 2 has a CDS of 2562 bp (GenBank accession no. MK411809), encoding 853 amino acids. Compared to variant 1, variant 2 is missing a 60 bp fragment while the rest of the sequence is identical (Table 2). Compared with cattle, yak *KDM1A* has five nucleotide differences in the CDS (Table 3) but an identical amino acid sequence. The CDS of KDM1A gene variant 1 shares 93.29–99.81% sequence similarity with six animal species, namely, cattle (99.81%), wild yak (99.50%), sheep (97.75%), pig (93.77%), dog (93.29%), and horse (93.60%). Search for similarity between sequences was performed using NCBI BLAST.

The CDS of the *KDM4B* gene was 3351 bp in length (GenBank accession no. MH510242), coding 1116 amino acids. Compared with cattle, yak KDM4B lacks a segment of 25 amino acids in the middle of the sequence and 9 amino acids differ. All of the differences are shown in Table 4 and Table 5. The *KDM4B* gene sequence shares 87.74–99.07% identity with six animal species, namely, cattle (99.07%), wild yak (97.94%), sheep (96.84%), pig (90.95%), dog (88.46%), and horse (87.74%), suggesting that the yak *KDM1A* and *KDM4B* genes are basically conserved.

### 3.3. KDM1A and KDM4B Expressions in the Testes of Yaks and Cattle–Yaks

**Figure 2:**
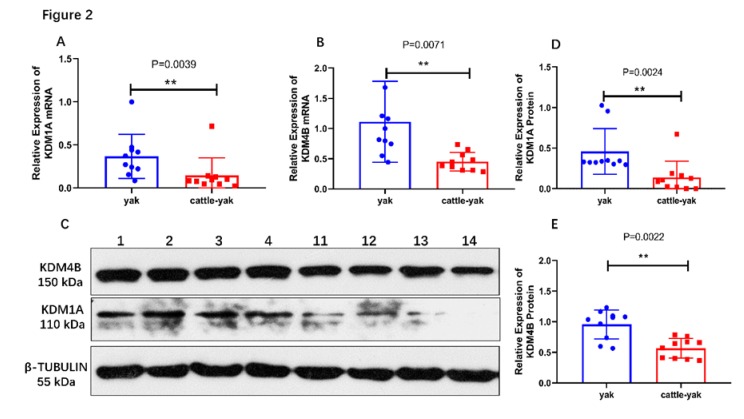
*KDM1A* and *KDM4B* expression in the testes of yaks and cattle–yaks. (**A**) The relative expression of the *KDM1A* mRNA was detected by Real-time PCR; GAPDH and 18S RNA were used as reference genes. Test for normal distribution (P_yak_ = 0.0651, P_cattle-yak_ < 0.0001). (**B**) The relative expression of the *KDM4B* mRNA was detected by Real-time PCR; GAPDH and 18S RNA were used as reference genes. Test for normal distribution (P_yak_ = 0.0534, P_cattle-yak_ = 0.3304). (**C**) The expression of KDM1A and KDM4B proteins was detected by Western blotting. β- Tubulin was used as reference gene. Lane 1–4 are yaks and lane 11-14 are cattle-yaks. (**D**) The relative expression of the KDM1A protein between yaks and cattle–yaks. Test for normal distribution (P_yak_ < 0.0001, P_cattle-yak_ = 0.0005). (**E**) The relative expression of the KDM4B protein between yaks and cattle–yaks. Test for normal distribution (P_yak_ = 0.0509, P_cattle-yak_ = 0.2195). ** *p* < 0.01.

The mRNA levels of *KDM1A/KDM4B* in the testes of yaks and cattle–yaks were assayed using quantitative Real-time PCR with *KDM1A/KDM4B*-specific primers. The results showed that the relative mRNA levels of the *KDM1A* and *KDM4B* genes in the testes of cattle–yaks were significantly lower (*p* < 0.01) than those in yaks (Figure 2A,B).

The expressions of KDM1A and KDM4B proteins in testes were tested by Western blotting (Figure 2C–E). The results showed the expressions of the KDM1A and KDM4B proteins in cattle–yaks were also significantly lower than those in yaks (Figure 2D,E).

### 3.4. The Levels of H3K36me3 and H3K27me3 in the Testes of Yaks and Cattle–Yaks

**Figure 3:**
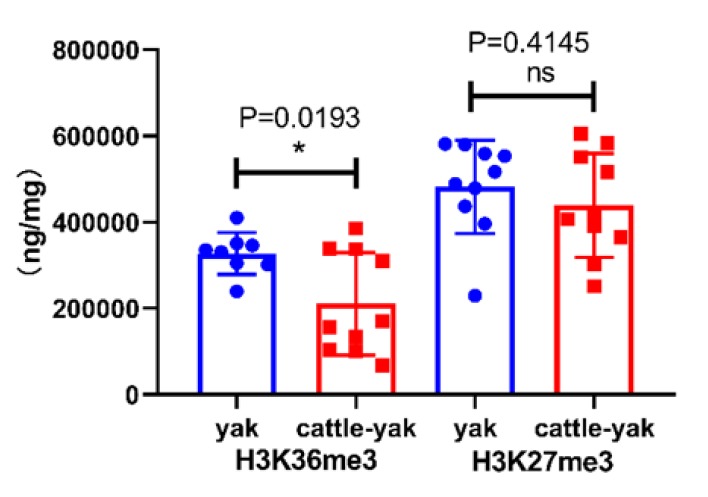
The levels of H3K36me3 and H3K27me3 in the testes of yaks and cattle-yaks. * *p* < 0.05. Test for normal distribution of H3K36me3 (P_yak_ = 0.5018, P_cattle-yak_ = 0.0797); Test for normal distribution of H3K27me3 (P_yak_ = 0.1168, P_cattle-yak_ = 0.6950).

The ELISA results showed that the level of H3K36me3 was significantly decreased in the testes of cattle–yaks compared with yaks, but not for H3K27me3 (Figure 3).

## 4. Discussion

To date, the molecular mechanism for male sterility of cattle–yaks has been unclear. Histological examination showed that the number of germ cells was significantly decreased in seminiferous tubules of cattle–yaks compared with yaks [4]. Spermatogenesis has been reported to be blocked in the primary spermatocyte stage [2,28]. A previous study found that few germ cells developed further than the stage of pachytene spermatocytes [29]. During meiosis, only a few autosomes of spermatocytes can form the synaptonemal complex (SC) [4]. In this study, a large amount of sperm was found in the epididymal ducts of the yaks. However, in the sections corresponding to the cattle–yaks, sperm was absent in the epididymal duct. This result was consistent with previous studies [2].

Comparing yaks and cattle, *KDM1A* codes for exactly the same amino acids in the CDS region but uses a different synonymous codon in five places. Codon usage bias represents unique feature of living things. Synonymous codons can affect mRNA stability, mRNA biogenesis, and even protein expression [30,31,32]. The demethylase enzymes precisely regulate the methylation status of histone lysine residues [24,33]. Histone methylation was originally thought to be an irreversible modification until the discovery of the first demethylase, KDM1A. KDM1A can specifically demethylate H3K4me1/2 and H3K9me1/2, which are respectively involved in activating and repressing transcription [21,34,35]. Evidence shows that *KDM1A* is implicated in the differentiation pathways for plasma, hematopoietic, adipogenesis, skeletal muscle, and embryonic stem cells [36,37,38,39,40]. Similarly, it plays an important role during spermatogenesis and differentiation of spermatogonial stem cells (SSCs) [38]. In this study, the expression levels of the mRNA and protein of *KDM1A* in the testes of cattle–yaks were significantly lower than those in yaks. *KDM1A* has been shown to play important roles in spermatogenesis and differentiation. Reduced levels of KDM1A lead to changes in the expression of a large number of genes associated with the maintenance and differentiation of spermatogonial stem cells [38]. A low level of *KDM1A* may lead to spermatocyte apoptosis and hindered spermatogonial differentiation [41]. A low level of KDM1A may be related to male sterility in cattle–yaks. The relationship between *KDM1A* and cattle–yak sterility deserves further study.

The KDM4 family consists of proteins KDM4A to KDM4D, which are H3K9me2/H3K9me3 and H3K36me2/H3K36me3 histone demethylases [42,43,44,45]. We cloned the CDS of yak *KDM4B*, which lacks 25 amino acids compared to the CDS of the bovine *KDM4B* gene. Compared with cattle, the *KDM4B* of yaks had nine amino acid alterations. It is unclear whether this may affect the gene function. The missing 25 amino acids are also lacking in the *KDM4B* sequences of both wild yaks and zebus when using the *KDM4B* CDS of wild yaks (XM_014482676.1) and zebus (XM_019964409.1) for comparison. The histone lysine demethylase KDM4B is related to the processes of DNA damage, embryonic stem cell reprogramming, and somatic cell reprogramming into pluripotency [45,46,47]. KDM4B plays a critical role in somatic cell reprogramming into pluripotency and osteogenic differentiation of human mesenchymal stem cells by reducing H3K9me3 and H3K36me3 levels [47,48]. The characterization of mRNA and protein levels of *KDM4B* showed that the levels in cattle–yaks were significantly lower than those in yaks. To understand the significance of the decrease in *KDM4B*, we compared the level of H3K36me3 between yaks and cattle–yaks.

The function of H3K36me3 is related to transcriptional activation [49]. In related studies, the level of H3K36me3 plays important roles in germ cell differentiation, somatic cell reprogramming, and chromosome recombination [25,47,50]. It was reported that normal methylation levels of H3K36me3 are required for spermatogenesis and sperm differentiation [25]. The localization of H3K36me3 in mouse spermatocytes indicates that H3K36me3 plays an important role in meiotic recombination [50]. This view is also confirmed by the decrease in homologous recombination chromosomal repair events as the level of H3K36me3 decreases [51]. In the present study, levels of H3K36me3 in cattle–yaks were significantly lower than those in yaks. Low levels of H3K36me3 have probably repressed meiosis and blocked germ cell differentiation in cattle–yaks [25,50]. Levels of KDM4B and H3K36me3 were both lower in cattle–yaks compared with yaks, and these results do not appear to be consistent with findings that the levels of H3K36me3 were significantly decreased after inducible expression of KDM4B [47]. The levels of H3K36me3 in cattle–yaks may be related to the methyltransferase Prdm9. Prdm9, acting as a methyltransferase, can methylate the trimethylation of H3K4 and H3K36 on the same nucleosome and, importantly, H3K4 and H3K36 double-positive nucleosomes only appear in the recombination region [50]. In our previous study, we found that the expression level of *Prdm9* in cattle–yaks was significantly lower than that in yaks [2]. We then combined those results with previously published studies to speculate that the reduction of *Prdm9* in cattle–yaks may have reduced the level of H3K36me3, and this may be inhibiting meiosis in cattle–yak spermatogenic cells [50].

H3K27me3 serves as a marker for gene repression and is pivotal for the formation of facultative heterochromatin and the repression of important transcriptional regulators during development [49,52]. H3K27me3 inhibits gene expression, thus maintaining the totipotency of stem cells [53]. Compared with yaks, the level of H3K27me3 was no significant difference in cattle–yaks testes. Research has shown that H3K27me3 is abundant in spermatogonial cells [12]. However, there were few spermatogonium cells in cattle–yak testicles, suggesting that the lack of difference in H3K27me3 between yaks and cattle–yaks is probably because of the antagonistic effect of H3K36me3 on H3K27me3 [12].

## 5. Conclusions

This study showed that the mRNA and protein levels of *KDM1A* and *KDM4B*, as well as H3K36me3, in sterile cattle–yak testes were significantly lower than in those of yaks. The decreased level of testicular H3K36me3 in cattle–yaks might be related to their sterility. The results provide epigenetic information underlying the molecular mechanism of cattle–yak male sterility. However, the testes include many types of cells such as Spermatogonia, spermatocytes and spermatids at different stages, Sertoli cells, Leydig cells. It is not enough to only examine the expression levels in testis tissue. The expression levels of KDM1A, KDM4B and H3K36me3 in specific sorted cells should be examine. Therefore, the results of this study have certain limitations, which will become the focus of our subsequent research.

## Figures and Tables

**Table 1:** Primers and conditions used for cloning and Real-time PCR.

GenBank Accession No.	Gene Name	Primer Sequence (5′–3′)	Annealing Temperature (°C)	Product Size (bp)	Application
XM_005203319.2	*KDM1A*	F: CCATGGAAACGGGAATCGCAG R: ACGTCTGTCTCACATGCTCG	59	2342	CDS clone
XM_005203319.2	*KDM1A*	F: GCGGACTGTGTGAAGAGAG R: CTCGTCTTCTGAGAGGTTGG	60	497	CDS clone
MK411808	*KDM1A*	F: TGTGCTTGTCCACCGAGT R: CCTGGCTTCCAGAAGTGTG	60	193	Real-time PCR
XM_014482676.1	*KDM4B*	F: TGACAAGGAACCGTGAAGTGT R: ATTCACAGCAAGCAAACGCCAG	59	3467	CDS clone
MH510242	*KDM4B*	F: GTGGCCTACATCGAGTCC R: GTGCAGTACTTCTCGCTG	60	233	Real-time PCR
NM 001034034	*GAPDH*	F: CGACTTCAACAGCGACACTCA R: GGTCCAGGGACCTTACTCCTT	60	169	Real-time PCR
NR 036642	*18S RNA*	F: CTGAGAAACGGCTACCACATC R: CAGACTTGCCCTCCAATGG	59	168	Real-time PCR

Note: F: forward primer; R: reverse primer.

**Table 2:** Nucleotide differences in the coding sequences of bovine and yak *KDM1A* genes.

Species	Variant	Nucleotide Position					
		21	36	520-579	1431	1524	1860
Cattle	1	G	G	G-A	G	A	C
Cattle	2	G	G	-	G	A	C
Yak	1	A	T	G-A	A	G	G
Yak	2	A	T	-	A	G	G

Note: Nucleotide position refers to the predicted bovine KDM1A (XM_005203319.2). 520-579: GGGCAAGCAGGAGGACTTCAAGACGACAGTTCTGGAGGGTATGGAGACGGCCAAGCATCA.

**Table 3:** Amino acid differences in the coding sequences of bovine and yak KDM1A proteins.

Species	Variant	Amino Acid Position
		174–193
Cattle	1	GlyGlnAlaGlyGlyLeuGlnAspAspSerSerGlyGlyTyrGlyAspGlyGlnAlaSer
Cattle	2	—
Yak	1	GlyGlnAlaGlyGlyLeuGlnAspAspSerSerGlyGlyTyrGlyAspGlyGlnAlaSer
Yak	2	—

Note: Amino acid position refers to the predicted bovine KDM1A (XM_005203319.2).

**Table 4:** Nucleotide differences in the coding sequences of bovine and yak *KDM4B* genes.

**Species**	**Nucleotide Position**												
	147	278	372	390	399	507	778	836	846	1101	1221	1295	1668
Cattle	T	T	G	A	T	C	C	G	T	T	A	T	C
Yak	C	C	A	G	C	T	T	A	C	C	T	C	T
**Species**	**Nucleotide Position**												
	1686	1827	2057	2078	2084	2169	2220	2262	2292	2399	2576	2706	2769
Cattle	A	T	A	T	T	T	T	G	C	A	A	T	T
Yak	G	C	G	C	C	C	C	A	T	G	G	C	C
**Species**	**Nucleotide Position**												
	2797–2871											2979	3162
Cattle	GTGAGTGCCCGTCTGCCCCACAGTCTGTTCCCCGGCCCCG CTGTCCTGCTGTGTTCTCATCCCCTCCACCTGCAG											C	G
Yak	—											T	A

Note: Nucleotide position refers to the predicted bovine KDM4B (XM_024994995.1).

**Table 5:** Amino acid differences in the coding sequences of bovine and yak KDM4B.

Species	Amino Acid Position									
	93	260	279	432	686	693	695	800	859	933–957
Cattle	Met	Arg	Gly	Val	Asn	Phe	Val	Asn	Asp	Val–Gln
Yak	Thr	Trp	Asp	Ala	Ser	Ser	Ala	Ser	Gly	—

Note: Amino acid position refers to the predicted bovine KDM4B (XM_024994995.1). 933–957: Val Ser Ala Arg Leu Pro His Ser Leu Phe Pro Gly Pro Ala Val Leu Leu Cys Ser His Pro Leu His Leu Gln.
